# Improving sensitivity of autoantibody testing for myasthenia gravis using cell-based assays: an evaluation of strategies that may be used in clinical practice

**DOI:** 10.3389/fneur.2026.1874030

**Published:** 2026-07-03

**Authors:** Adrian Budhram, Ola Z. Ismail, Kamala Sangam, Lulu L. C. D. Bursztyn, Ario Mirian, Yiu-Chia Chang, Michael W. Nicolle

**Affiliations:** 1 Department of Clinical Neurological Sciences, Western University, London Health Sciences Centre, London, ON, Canada; 2 Department of Pathology and Laboratory Medicine, Western University, London Health Sciences Centre, London, ON, Canada; 3 Department of Ophthalmology, Western University, St. Joseph’s Health Care London, London, ON, Canada; 4 Department of Medicine, University of Toronto, St. Michael’s Hospital, Toronto, ON, Canada

**Keywords:** acetylcholine receptor antibody, autoantibody testing, cell-based assay, muscle-specific kinase antibody, myasthenia gravis

## Abstract

**Introduction:**

Testing for acetylcholine receptor and muscle-specific kinase antibodies (anti-AChR and anti-MuSK) is of central diagnostic importance in myasthenia gravis (MG), yet practical evaluations of strategies to improve sensitivity using cell-based assays (CBAs) are lacking. We evaluated strategies to improve sensitivity of autoantibody testing for MG using CBAs at our center, including repeat fixed CBA at routine 1:10 dilution, send-out live CBA, and fixed CBA at lower 1:5 dilution.

**Methods:**

Quality improvement/quality assurance (QI/QA) evaluation of all patients at London Health Sciences Centre/St. Joseph’s Health Care London (LHSC/SJHC) who underwent anti-AChR/MuSK fixed CBA testing from January 2023–January 2026.

**Results:**

During the 3-year evaluation period, 973 patients underwent anti-AChR/MuSK fixed CBA at routine 1:10 dilution. Of these, 127 (13.1%) exhibited autoantibody positivity, 3 (0.3%) were indeterminable, and 843 (86.6%) were negative. Testing of 78 patients represented repeat testing after initially negative anti-AChR/MuSK fixed CBA, of whom 4 (5%) were positive. Thirteen patients with negative/indeterminable anti-AChR/MuSK fixed CBA underwent send-out live CBA, of whom 4 (31%) were positive. Of all 131 anti-AChR/MuSK-positive patients identified, 123 (94%) had positivity on initial fixed CBA, 4 (3%) on repeat fixed CBA, and 4 (3%) on send-out live CBA. Among 8 samples with negativity by fixed CBA at 1:10 dilution from patients with subsequent autoantibody positivity, 4 (50%) exhibited positivity at 1:5 dilution.

**Conclusion:**

Repeat fixed CBA at routine 1:10 dilution, send-out live CBA, and fixed CBA at lower 1:5 dilution are all strategies that may improve sensitivity of autoantibody testing for MG at our center.

## Introduction

Testing for acetylcholine receptor and muscle-specific kinase antibodies (anti-AChR and anti-MuSK) is a cornerstone of myasthenia gravis (MG) evaluation. Anti-AChR/MuSK fixed cell-based assay (CBA) has demonstrated excellent specificity as well as higher overall sensitivity than radioimmunoassay (RIA), and is used in our laboratory as first-line testing (1, 2). However, use of multiple assays has been suggested to maximize sensitivity in persistently suspicious cases of MG, and in particular live CBA may detect autoantibodies that are missed by RIA or fixed CBA (2–4). At many centers including our own, there are practical limitations to use of live CBA as first-line testing; however, in January 2023 we developed a formalized live CBA send-out process for patients with negative anti-AChR/MuSK fixed CBA but persistently high suspicion for MG to improve sensitivity. Other strategies reported to improve sensitivity include repeat testing by the same methodology (5), and performing fixed CBA at a lower serum dilution of 1:5 (instead of 1:10 as per manufacturer’s instructions) (6). We opted to perform a quality improvement/quality assurance (QI/QA) evaluation of these strategies prior to their potential incorporation into our autoantibody testing algorithm for MG.

## Methods

We identified all patients at London Health Sciences Centre/St. Joseph’s Health Care London (LHSC/SJHC) who underwent serum anti-AChR (including anti-AChR-E [adult-type] and anti-AChR-G [fetal-type]) and anti-MuSK fixed CBA (Euroimmun) at routine 1:10 dilution from January 2023–January 2026 like described previously (1). For each patient, AChR-E, AChR-G and MuSK fixed CBA were reported as Negative, Weak Positive or Positive by two independent readers (Adrian Budhram with 4.5 years experience reading anti-AChR/MuSK fixed CBA on a clinical service basis; Ola Z. Ismail with 1.5 years experience reading anti-AChR/MuSK fixed CBA on a clinical service basis) using manual microscopy (Olympus BX40), with discussion to achieve consensus in discrepant cases. Weak Positive refers to staining that is faint but of sufficient intensity above the background to suggest a positive result, as described previously (1, 7). A patient with a Weak Positive or Positive result reported for AChR-E, AChR-G or MuSK fixed CBA was considered to have a positive autoantibody result.

Among patients with initially negative/indeterminable fixed CBA, those who underwent repeat anti-AChR/MuSK fixed CBA or send-out anti-AChR/MuSK/LRP4 live CBA (Oxford University) were identified, and the proportion with positive results was calculated to investigate the yield of these strategies. Our center has no restrictions specific to ordering repeat fixed CBA, but ordering send-out live CBA requires review by an MG specialist (Michael W. Nicolle) to confirm persistently high suspicion for MG (typically generalized MG, given the higher frequency and greater therapeutic impact of autoantibody positivity compared to ocular MG). An aliquot of serum being sent out for live CBA is first re-tested by fixed CBA to confirm negativity prior to shipment.

To investigate the yield of fixed CBA at 1:5 dilution, samples that were initially negative/indeterminable by fixed CBA at 1:10 dilution but considered to be suspicious for autoantibody positivity (i.e., samples from patients with subsequent autoantibody positivity on repeat fixed CBA at 1:10 dilution, or samples sent out for live CBA) were retrieved from −80 °C storage and re-tested at 1:5 dilution.

Median turnaround time (TAT) of send-out live CBA versus in-house fixed CBA was compared by Mann–Whitney U Test, and per-sample cost of send-out live CBA (including shipping) relative to in-house fixed CBA was determined.

As per our institutional research ethics board (REB), QI/QA evaluations do not fall within the scope of institutional ethical review under Article 2.5 of the Tri-Council Policy Statement: Ethical Conduct for Research Involving Humans (TCPS 2). While not requiring REB review, ethical issues that may arise during QI/QA evaluations of laboratory testing at LHSC are still thoroughly considered within the Department of Pathology and Laboratory Medicine to minimize any potential risk of harm to participants and ensure that ethical requirements for the protection of human participants in Quality Assurance/Improvement are met (8, 9).

## Results

### Repeat fixed CBA and send-out live CBA each captured anti-AChR/MuSK-positive patients that were missed by initial fixed CBA

During the 3-year evaluation period, 973 patients underwent anti-AChR/MuSK fixed CBA. Of these, 127 (13.1%) exhibited autoantibody positivity (anti-AChR, 124; anti-MuSK, 3), 3 (0.3%) were indeterminable (high background fluorescence, 2; equivocal fluorescence of anti-AChR-E/G wells compared to the control well, 1), and 843 (86.6%) were negative. Testing of 78 patients (which included those being re-tested by fixed CBA to confirm negativity prior to send-out live CBA) represented repeat testing of follow-up samples after initially negative anti-AChR/MuSK fixed CBA; among these patients, repeat fixed CBA identified a positive autoantibody in 4/78 (5%). These 4 patients, along with 1 patient who was positive on repeat fixed CBA that was prompted by fixed CBA results at 1:5 dilution (discussed later), are summarized in Table 1. At time of initial fixed CBA, 4/5 (80%) exhibited ocular symptoms only and 3/5 (60%) had symptom onset within 1 month.

**Table 1:** Summary of patients with initially negative/indeterminable anti-AChR/MuSK fixed CBA results who were positive on repeat anti-AChR/MuSK fixed CBA testing.

Patient/demographic	Symptoms at time of initial negative fixed CBA testing	Time between symptom onset and initial negative fixed CBA testing	MG treatments at time of initial negative fixed CBA testing	MGFA at time of initial negative fixed CBA testing	Symptoms at time of positive repeat fixed CBA testing	Time between symptom onset and positive repeat fixed CBA testing	MG treatments at time of positive repeat fixed CBA testing	MGFA at time of positive repeat fixed CBA testing	Description of positive result on repeat fixed CBA testing	Evidence of thymic pathology?	Final diagnosis	Result of fixed CBA testing at 1:5 dilution, using sample that was initially negative by fixed CBA
											Supportive evidence of MG*	
1/Middle-aged adult male**	Fluctuating diplopia	3 days	None	I	Fluctuating diplopia, ptosis	2.5 years	None	I	Positive for anti-AChR-E and positive for anti-AChR-G	No (CT thorax)	OMG	Weak positive for anti-AChR-E and weak positive for anti-AChR-G
		Again negative on repeat fixed CBA testing at 18 months									Fluctuating ocular muscle weakness, decrement on RNS	
2/Older adult female	Diplopia	5 days	None	I	Fluctuating diplopia, ptosis, dysphagia	7 months	None	IIIb	Positive for anti-AChR-E and positive for anti-AChR-G	No (CT thorax)	GMG	Positive for anti-AChR-E and positive for anti-AChR-G
											Fluctuating muscle weakness	
3/Older adult female	Fluctuating diplopia	8 months	None	I	Fluctuating diplopia, ptosis	19 months	Mestinon	I	Weak positive for anti-AChR-E and weak positive for anti-AChR-G	No (CT thorax)	OMG	Negative
											Fluctuating ocular muscle weakness	
4/Older adult female	Diplopia, UE weakness	4 weeks	None	IIIa	Diplopia, UE weakness	5 weeks	None	IIIa	Weak positive for anti-AChR-E	No (CT thorax)	GMG	Weak positive for anti-AChR-E
											Decrement on RNS	
5/Older adult male***	Fluctuating diplopia	2.5 years	None	I	None (diplopia resolved)	5.5 years	None	0	Positive for anti-AChR-E	No (CT thorax)	OMG	Positive for anti-AChR-E and positive for anti-AChR-G
											Fluctuating ocular muscle weakness	

AChR, acetylcholine receptor; CBA, cell-based assay; GMG, generalized myasthenia gravis; MG, myasthenia gravis; MGFA, myasthenia gravis foundation of America clinical classification; MuSK, muscle-specific kinase; OMG, ocular myasthenia gravis; RNS, repetitive nerve stimulation; UE, upper extremity. Age stratification is as follows: young adult, 19–45 years of age; middle-aged adult, 46–65 years of age; older adult, greater than 65 years of age. *In addition to fluctuating muscle weakness or decrement on RNS, all patients had no more likely alternative diagnosis identified. **This patient underwent fixed CBA testing a total of 3 times. Of the 2 initially negative fixed CBA samples (testing performed 3 days after symptom onset, and then 18 months after symptom onset), only the sample from testing performed 18 months after symptom onset exhibited positivity when re-tested at 1:5 dilution. ***Initial fixed CBA testing was indeterminable due equivocal fluorescence of anti-AChR-E/G wells compared to the control well, and negative on send-out live CBA. The finding of anti-AChR positivity by fixed CBA at 1:5 dilution prompted sample recollection 3 years after initial testing to perform repeat fixed CBA at routine 1:10 dilution, which was Positive for Anti-AChR-E. This recollected sample was again sent out for live CBA as a QI/QA measure, which returned Low Positive for Anti-AChR.

Thirteen patients with negative/indeterminable anti-AChR/MuSK fixed CBA results underwent send-out live CBA. Twelve negative samples were sent due to persistently high suspicion for MG (typically generalized MG), while 1 indeterminable sample (with equivocal fluorescence of anti-AChR-E/G wells compared to the control well) was sent by our laboratory as a QI/QA measure (for possible ocular MG). Send-out live CBA identified a positive autoantibody in 4/13 (31%). These 4 patients are summarized in Table 2. At time of initial fixed CBA, all 4 (100%) exhibited generalized symptoms.

**Table 2:** Summary of patients with initially negative anti-AChR/MuSK fixed CBA results who were positive on send-out live anti-AChR/MuSK/LRP4 CBA testing.

Patient/demographic	Symptoms at time of initial negative fixed CBA testing	Time between symptom onset and initial negative fixed CBA testing	MG treatments at time of initial negative fixed CBA testing	MGFA at time of initial negative fixed CBA testing	Symptoms at time of positive send-out live CBA testing	Time between symptom onset and positive send-out live CBA testing	MG treatments at time of positive send-out live CBA testing	MGFA at time of positive send-out live CBA testing	Description of positive result on send-out live CBA testing	Evidence of thymic pathology?	Final diagnosis	Result of fixed CBA testing at 1:5 dilution, using sample that was initially negative by fixed CBA
											Supportive evidence of MG*	
6/Young adult female	Fluctuating ptosis, facial weakness, dysphagia, dysarthria, UE and LE weakness	2.5 years	None	IIIb	Fluctuating ptosis, UE and LE weakness	4 years	Mestinon, prednisone	IIa	Low positive for anti-AChR	No (CT thorax)	GMG	Weak positive for anti-AChR-E
											Fluctuating muscle weakness, decrement on RNS	
7/Young adult female	Fluctuating diplopia, ptosis, dysarthria, dysphagia	8 months	None	IIIb	Fluctuating ptosis, dysarthria, dysphagia, UE and LE weakness	4 years	Mestinon, prednisone, azathioprine	IIIb	Low positive for anti-AChR	No (CT thorax)	GMG	Negative
											Fluctuating muscle weakness, decrement on RNS	
8/Older adult male	Fluctuating ptosis, facial weakness, dysarthria, dysphagia	15 months	None	IIIb	Fluctuating diplopia, ptosis, facial weakness, dysarthria, dysphagia	16 months	Mestinon, prednisone, IVIG	IVb	Low positive for anti-AChR and low positive for anti-MuSK	No (CT thorax)	GMG	Negative
											Fluctuating muscle weakness, decrement on RNS	
9/Middle-aged adult female	Fluctuating diplopia, LE weakness	10 months	None	IIIa	Fluctuating diplopia, LE weakness	18 months	Mestinon	IIIa	Low positive for anti-AChR	No (CT thorax)	GMG	Negative
											Fluctuating muscle weakness, decrement on RNS (as well as significant increment of >300%)	

AChR, acetylcholine receptor; CBA, cell-based assay; GMG, generalized myasthenia gravis; LE, lower extremity; LRP4, low-density lipoprotein receptor-related protein 4; MG, myasthenia gravis; MGFA, myasthenia gravis foundation of America clinical classification; MuSK, muscle-specific kinase; OMG, ocular myasthenia gravis; RNS, repetitive nerve stimulation; UE, upper extremity. Age stratification is as follows: young adult, 19–45 years of age; middle-aged adult, 46–65 years of age; older adult, greater than 65 years of age. *In addition to fluctuating muscle weakness or decrement on RNS, all patients had no more likely alternative diagnosis identified.

Of all 131 anti-AChR/MuSK-positive patients identified during the evaluation period, 123 (94%) had positivity identified on initial fixed CBA, 4 (3%) on repeat fixed CBA, and 4 (3%) on send-out live CBA.

### In some cases suspicious for autoantibody positivity, fixed CBA at 1:5 dilution revealed positivity that was missed at 1:10 dilution

We retrieved serum samples with sufficient quantity that were negative (*N* = 15) or indeterminable (*N* = 1) by fixed CBA at 1:10 dilution but considered to be suspicious for autoantibody positivity, and re-tested them by fixed CBA at 1:5 dilution. These included (a) serum samples that were negative by fixed CBA, from patients who exhibited positivity on repeat fixed CBA (*N* = 4), and (b) serum samples that were negative/indeterminable by fixed CBA, which were aliquoted from serum that was sent out for live CBA (*N* = 12). Of these 16 samples, fixed CBA was repeated at 1:10 dilution as well as 1:5 dilution in singlicate, interpretation blinded to patient identifiers was performed, and results at 1:5 dilution were compared to repeat fixed CBA or send-out live CBA results which served as the reference standard. Among 8 samples for which the reference standard was positive (4 by repeat fixed CBA, 4 by send-out live CBA), 4 (50%) exhibited positivity by fixed CBA at 1:5 dilution (Patients 1, 2, 4, and 6; see Tables 1, 2). Meanwhile, among 8 samples for which the reference standard was negative (by send-out live CBA), 1 (13%) exhibited positivity by fixed CBA at 1:5 dilution (Patient 5; see Table 1). On unblinded review, this patient was indeterminable on fixed CBA at routine 1:10 dilution due to equivocal fluorescence of anti-AChR-E/G wells compared to the control well, and negative on send-out live CBA. The finding of anti-AChR positivity by fixed CBA at 1:5 dilution prompted sample recollection 3 years after initial testing to perform repeat fixed CBA at routine 1:10 dilution, which was Positive for Anti-AChR-E (see Table 1). The reference standard for Patient 5 was therefore revised to positive. This recollected sample was again sent out for live CBA as a QI/QA measure, which returned Low Positive for Anti-AChR.

**Figure 1:**
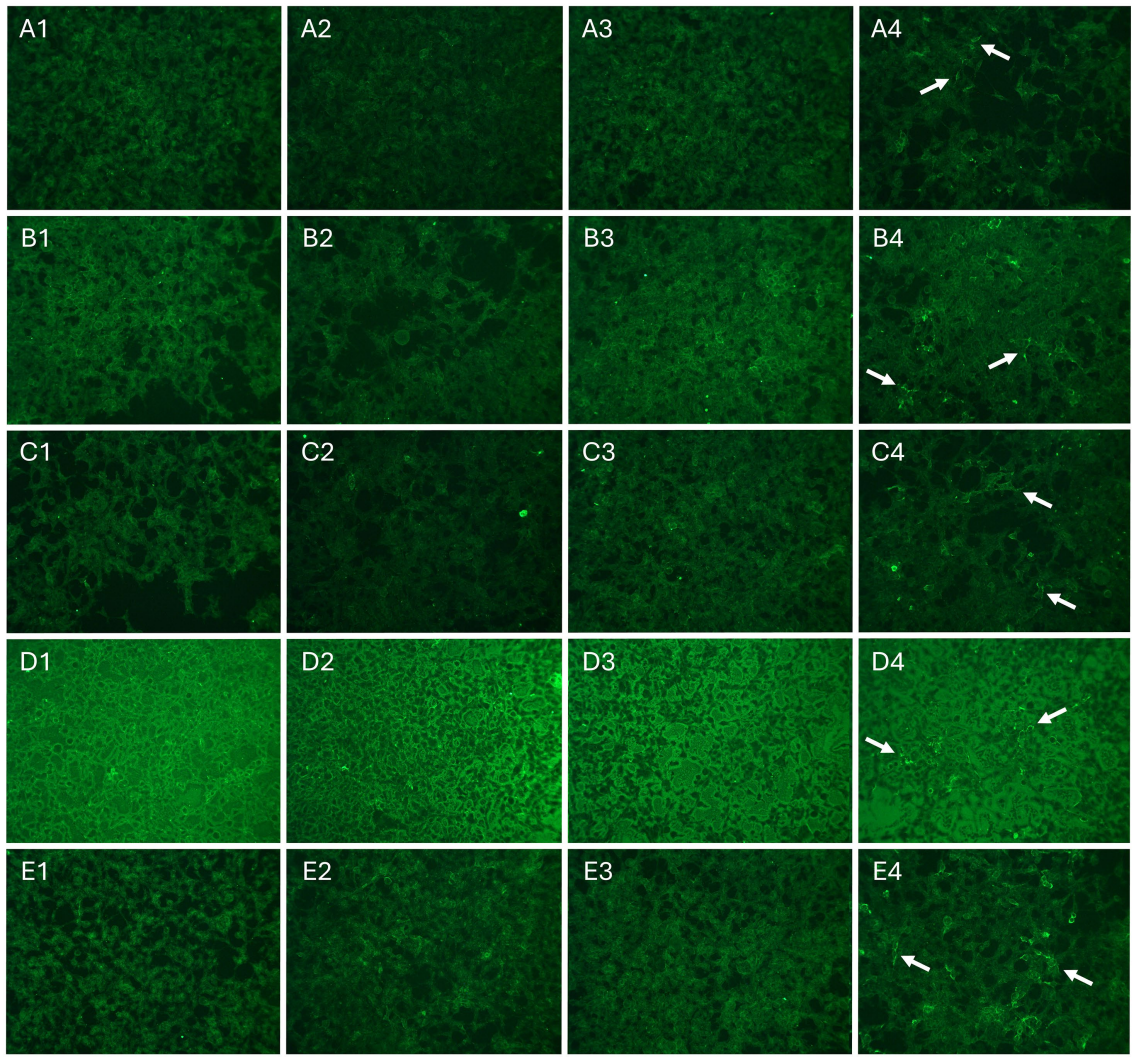
Positivity for Anti-AChR-E revealed by fixed CBA testing at 1:5 dilution. Fixed CBA testing at 1:5 dilution revealing positivity for anti-AChR-E in 5 patients (Patients 1, 2, 4, 5, and 6; see Tables 1, 2). Column 1 = Control well at 1:10 dilution; Column 2 = Anti-AChR-E well at 1:10 dilution; Column 3 = Control well at 1:5 dilution; Column 4 = Anti-AChR-E well at 1:5 dilution (arrows used to highlight areas of positivity). Row A = Patient 2; Row B = Patient 6; Row C = Patient 4; Row D = Patient 1; Row E = Patient 5. Patient 5 had equivocal fluorescence of the anti-AChR-E well compared to the control well on fixed CBA testing at routine 1:10 dilution (see manuscript text for additional details). All images taken at 20x magnification using INFINITY3 microscope camera mounted to Olympus BX40 manual microscope. AChR, acetylcholine receptor; CBA, cell-based assay.

Representative images of fixed CBA anti-AChR-E positivity revealed at 1:5 dilution compared to routine 1:10 dilution are shown in Figure 1.

### Compared to in-house fixed CBA, turnaround time was longer and cost was higher for send-out live CBA

The median TAT of send-out live CBA was significantly longer than in-house fixed CBA (40 days versus 3 days, *p* < 0.001), and the cost of send-out live CBA was approximately 4 times higher than that of in-house fixed CBA.

## Discussion

**Figure 2:**
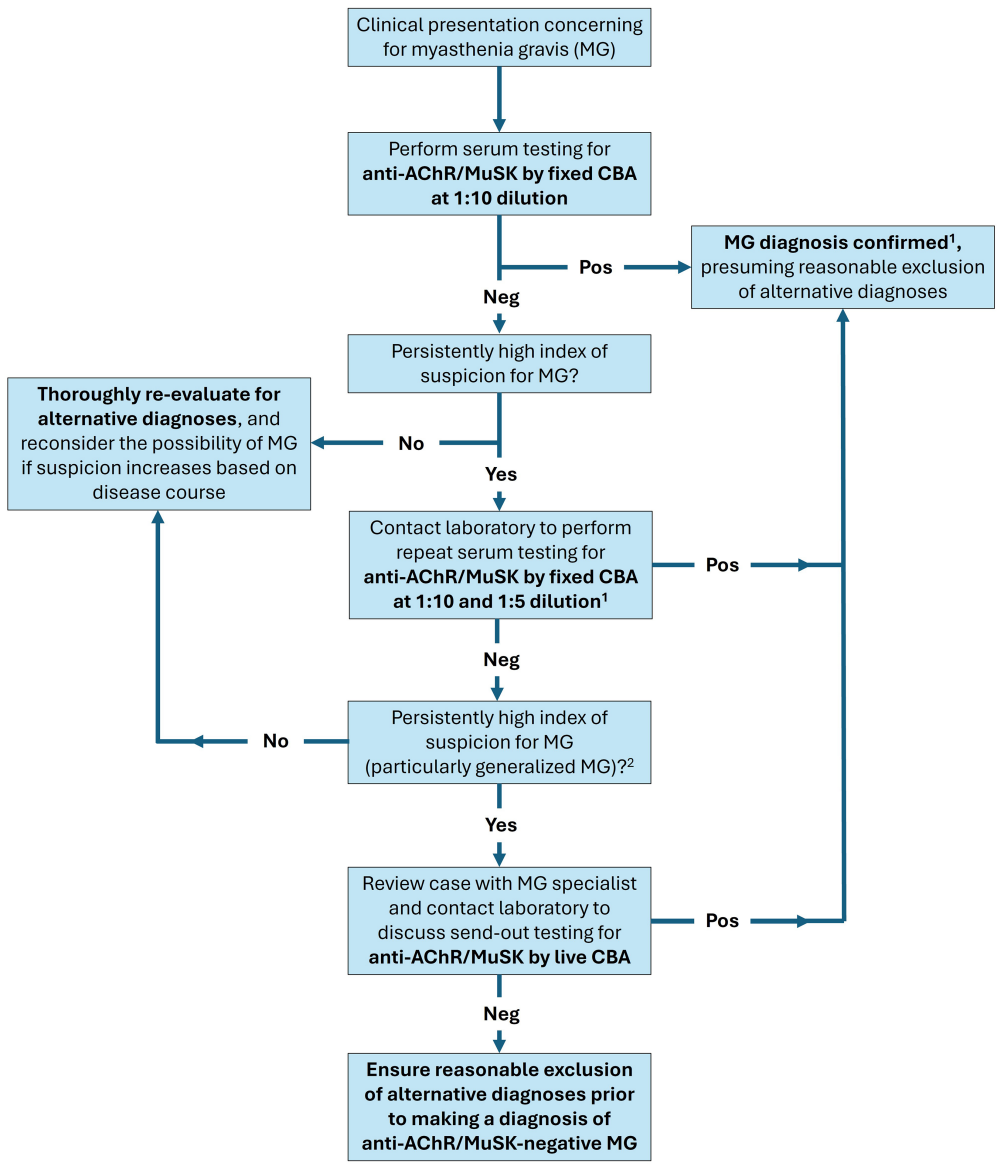
Autoantibody testing algorithm for MG using fixed CBA. (1) For repeat serum testing for anti-AChR/MuSK by fixed CBA that is negative at 1:10 dilution but positive at 1:5 dilution, confirmatory send-out live CBA will be performed as an ongoing QI/QA evaluation for the first year, followed by annual reassessment. This will serve to more robustly examine the specificity of fixed CBA at 1:5 dilution compared to the reference standard of send-out live CBA. Any discordant cases during this ongoing QI/QA evaluation (i.e., positive by fixed CBA at 1:5 dilution but negative by send-out live CBA) will undergo MG specialist assessment to facilitate diagnostic clarification. (2) Send-out live CBA testing at our institution is typically only performed for patients with a persistently high index of suspicion for generalized MG, given the higher frequency and greater therapeutic impact of autoantibody positivity compared to ocular MG. This serves to balance test yield and impact on clinical management with practical considerations such as TAT and cost. AChR, acetylcholine receptor; CBA, cell-based assay; MG, myasthenia gravis; MuSK, muscle-specific tyrosine kinase; QI/QA, quality improvement/quality assurance; TAT, turnaround time.

While the majority (94%) of anti-AChR/MuSK-positive patients at our institution were identified on initial fixed CBA, we found that repeat fixed CBA as well as send-out live CBA each captured additional patients (3% each). We also found that fixed CBA testing at a lower 1:5 dilution may reveal positivity that is missed at routine 1:10 dilution. We hypothesize that this reflects improved visualization of low-titer antibodies at a lower dilution, which is supported by autoantibody detection when using a different assay that may have higher analytical sensitivity (i.e., send-out live CBA) or using the same assay at routine 1:10 dilution later in the disease course (i.e., repeat fixed CBA at a later time point when antibody titers may be higher) in all these cases. Given our findings, for patients assessed at our center we plan to implement the autoantibody testing algorithm shown in Figure 2. This algorithm takes a tiered approach to testing that aims to improve sensitivity by using strategies that may increase yield in persistently suspicious cases of MG (repeat fixed CBA at routine 1:10 dilution, fixed CBA at lower 1:5 dilution, send-out live CBA), while balancing practical limitations of send-out live CBA that include longer TAT and higher cost.

Our evaluation has several limitations. Systematic comparison of sensitivity and specificity of the different testing strategies was outside the scope of this evaluation, which aimed to explore their potential to improve sensitivity in our practice setting. In particular, robust evaluation of the specificity of fixed CBA at lower 1:5 dilution was not performed as part of this evaluation; for this reason, in a patient who is negative at 1:10 dilution but positive at 1:5 dilution using fixed CBA at our institution moving forward, confirmatory send-out live CBA will still be performed as an ongoing QI/QA evaluation (see Figure 2). The potential impact of immunotherapy on test sensitivity among all patients who tested negative was not assessed as part of this evaluation; however, no patient with positivity identified by repeat fixed CBA or send-out live CBA was on immunotherapy at time of initially negative fixed CBA testing, indicating that immunotherapy was not a contributor to their initially negative autoantibody result. A clinical reference standard was not uniformly applied against all autoantibody results, like has been done previously in evaluations dedicated to determination of test sensitivity and specificity; however, all patients with autoantibody positivity identified by the strategies evaluated herein (repeat fixed CBA at routine 1:10 dilution, fixed CBA at lower 1:5 dilution, send-out live CBA) were confirmed to have clinical or electro-diagnostic features supportive of MG and no more likely alternative diagnosis identified, which is supportive of true-positivity (see Tables 1 and 2). Ordering send-out live CBA at our institution requires review by an MG specialist to confirm persistently high suspicion for MG (typically generalized MG); while this is meant to balance yield with practical considerations of send-out testing, there is an element of subjectivity to this process and it is likely to result in enrichment of the tested population, which may overestimate the yield of send-out live CBA at our center. A majority of patients with autoantibody positivity identified on repeat fixed CBA at routine 1:10 dilution had symptom onset within 1 month of initial fixed CBA testing and presented with ocular symptoms only; this suggests that repeat fixed CBA may be of particular benefit in patients with persistent concern for MG who initially test negative when their symptoms are relatively mild and they are early in their disease course, similar to previous work highlighting that initially seronegative MG patients may later become seropositive (10). However, small numbers and differing stringency in ordering processes for repeat fixed CBA versus send-out live CBA at our center preclude formal comparative analysis by testing strategy, which would benefit from further evaluation. Fixed CBA interpretation at 1:5 dilution was blinded to patient identifiers, but reader awareness of reported improved sensitivity at this dilution could still result in interpretative bias; representative images are included as a figure to mitigate this potential concern. Our institution no longer performs or sends out autoantibody testing by RIA, given reported overall lower sensitivity and possibly lower overall specificity compared to fixed CBA; for this reason, it was not evaluated for potential incorporation in our algorithm (1, 2, 11). Recent literature has suggested that RIA may detect low-concentration anti-AChR that is missed by fixed CBA testing at 1:10 dilution (12), although fixed CBA testing at 1:5 dilution as well as send-out live CBA testing like we have included in our autoantibody testing algorithm would be expected to help capture such cases. Interpretation of fixed CBA is subject to reader expertise, so our findings may not be generalizable to less experienced centers. Our determinations of TAT and relative cost may similarly not be generalizable to other practice settings (e.g., clinical laboratories that perform RIA or live CBA in-house), although our findings highlight the importance of examining these variables when developing local autoantibody testing algorithms. Finally, only patients who underwent send-out live CBA were tested for anti-LRP4 and none exhibited positivity for this antibody; although the number of patients tested for this antibody was small, the lack of yield of anti-LRP4 live CBA testing in our evaluation is in line with recent work (13).

Our evaluation indicates that repeat fixed CBA at 1:10 dilution, at 1:5 dilution, and send-out live CBA are all strategies that can increase yield in patients with persistent suspicion for MG. Incorporation of these strategies into our local autoantibody testing algorithm aims to improve sensitivity while balancing practical limitations, and enhance the quality of diagnostic service we are able to offer for patients with MG at our center.

Disclosures: Adrian Budhram reports that he holds the London Health Sciences Centre and London Health Sciences Foundation Chair in Neural Antibody Testing for Neuro-Inflammatory Diseases. Ola Ismail has no relevant disclosures to report. Kamala Sangam has no relevant disclosures to report. Lulu Bursztyn has no relevant disclosures to report. Ario Mirian has no relevant disclosures to report. Yiu-Chang has no relevant disclosures to report. Michael Nicolle has no relevant disclosures to report.

## Data Availability

The raw data supporting the conclusions of this article will be made available by the authors, without undue reservation.
